# A distinct intra-individual suppression subnetwork in the brain’s default mode network across cognitive tasks

**DOI:** 10.1093/cercor/bhac361

**Published:** 2022-09-20

**Authors:** Christine A Leonards, Ben J Harrison, Alec J Jamieson, Trevor Steward, Silke Lux, Alexandra Philipsen, Christopher G Davey

**Affiliations:** Melbourne Neuropsychiatry Centre, Department of Psychiatry, The University of Melbourne, Parkville, Victoria, 3010, Australia; Melbourne Neuropsychiatry Centre, Department of Psychiatry, The University of Melbourne, Parkville, Victoria, 3010, Australia; Melbourne Neuropsychiatry Centre, Department of Psychiatry, The University of Melbourne, Parkville, Victoria, 3010, Australia; Melbourne Neuropsychiatry Centre, Department of Psychiatry, The University of Melbourne, Parkville, Victoria, 3010, Australia; Melbourne School of Psychological Sciences, The University of Melbourne, Parkville, Victoria, 3010, Australia; Department of Psychiatry and Psychotherapy, University of Bonn, Bonn, 53127, Germany; Department of Psychiatry and Psychotherapy, University of Bonn, Bonn, 53127, Germany; Department of Psychiatry, The University of Melbourne, Parkville, Victoria, 3010, Australia

**Keywords:** cognition, deactivation, default mode network, fMRI, individual differences

## Abstract

Suppression of the brain’s default mode network (DMN) during external goal-directed cognitive tasks has been consistently observed in neuroimaging studies. However, emerging insights suggest the DMN is not a monolithic “task-negative” network but is comprised of subsystems that show functional heterogeneity. Despite considerable research interest, no study has investigated the consistency of DMN activity suppression across multiple cognitive tasks within the same individuals. In this study, 85 healthy 15- to 25-year-olds completed three functional magnetic resonance imaging tasks that were designed to reliably map DMN suppression from a resting baseline. Our findings revealed a distinct suppression subnetwork across the three tasks that comprised traditional DMN and adjacent regions. Specifically, common suppression was observed in the medial prefrontal cortex, the dorsal-to-mid posterior cingulate cortex extending to the precuneus, and the posterior insular cortex and parietal operculum. Further, we found the magnitude of suppression of these regions were significantly correlated within participants across tasks. Overall, our findings indicate that externally oriented cognitive tasks elicit common suppression of a distinct subnetwork of the broader DMN. The consistency to which the DMN is suppressed within individuals suggests a domain-general mechanism that may reflect a stable feature of cognitive function that optimizes external goal-directed behavior.

## Introduction

The discovery of the brain’s default mode network (DMN) is one of the most remarkable achievements of neuroimaging. Anatomically, the network comprises the medial prefrontal cortex (MPFC), posterior cingulate cortex (PCC), and the angular gyrus region of the inferior parietal cortex (IPL; Raichle et al. 2001; Buckner et al. 2008; Andrews-Hanna et al. 2010). The DMN is characterized by relatively high resting activity that is “suppressed” during externally directed tasks, reverting back to this “default mode” when the focus on external stimuli is relaxed (Shulman et al. 1997; Raichle et al. 2001; Buckner et al. 2008). Consistent with this dynamic activity, the DMN has been shown to support internal self-referential processes (Raichle et al. 2001; Harrison et al. 2008; Spreng and Grady 2010; Davey et al. 2016), which are suspended when attention is oriented externally in order to optimize task-related performance (Shulman et al. 1997; Raichle et al. 2001; Buckner et al. 2008). Hence, DMN suppression is thought to represent an important adaptive feature of brain function vital for efficient cognitive functioning; facilitating the transfer of attentional resources between internal and external mental states (Gusnard et al. 2001; Raichle et al. 2001; Crittenden et al. 2015; Smith et al. 2018).

Consistent with this view, DMN suppression has been observed across many cognitive tasks (Shulman et al. 1997; Binder et al. 1999; Mazoyer et al. 2001) with the magnitude of suppression increasing with general task demand (McKiernan et al. 2003; McKiernan et al. 2006; Singh and Fawcett 2008; Harrison et al. 2011). Such observations suggest that cognitive resources used for internal processes are progressively attenuated, or disengaged, to facilitate externally oriented cognitive processing. The anatomical consistency with which task-induced DMN suppression has been observed across different tasks and studies has led to the notion that it is a task-independent phenomenon reflecting a homogenous “task-negative” network (Fox et al. 2005).

However, challenging this “task-negative” hypothesis, some studies have found selective variability in the nature of DMN suppression in response to changing levels of task demand. For instance, Mayer et al. (2010) modulated cognitive load across working memory and visual attention tasks and observed the two tasks elicited differential suppression at higher cognitive loads in the MPFC, medial parietal (i.e. PCC, precuneus) and lateral temporal cortices. In a meta-analysis of five imaging tasks, Wicker et al. (2003) identified common suppression during high demand conditions only in the dorsal MPFC, while McKiernan et al. (2003) found common suppression during three demanding auditory conditions only in the anterior cingulate cortex (ACC). Interestingly, Harrison et al. (2011) observed that, at higher levels of task demand, activity suppression also extended to non-DMN regions, including the posterior insula cortex and parietal operculum.

Together, the above evidence suggests two key features of DMN suppression: first, that the network may be subdivided into regions that show suppression irrespective of task type and regions that show preferential suppression dependent on the nature of the task; and second, that task-induced suppression is not limited to traditionally recognized DMN regions. This view aligns with more recent conceptualizations of the DMN that suggest it is not a unitary network that suppresses as a uniform entity as a function of internally- versus externally-directed mental processes (Leech et al. 2011; Fornito et al. 2012; Leech and Sharp 2014) but is a complex network comprised of subsystems that show functional heterogeneity (Andrews-Hanna et al. 2010; Buckner and DiNicola 2019). However, despite considerable ongoing research interest in the DMN, no study has investigated its suppression across multiple cognitive tasks in the same participants. Thus, the anatomical and functional consistency of DMN suppression and the significance of these potentially selective suppression effects remain unclear.

The aim of this study was to investigate task-induced modulation of DMN suppression across multiple externally oriented attentionally demanding cognitive tasks involving different cognitive functions and to determine the consistency of this suppression effect within individuals. In doing so, we sought to make stronger conclusions regarding the anatomical (i.e. spatial) and functional (i.e. magnitude) consistency of DMN suppression during cognitive task performance. As a secondary aim, we sought to investigate whether any individual differences (i.e. age, IQ, or ruminative style) were associated with individual characteristics. To do this, we recruited a large sample of healthy individuals who completed three distinct, commonly used imaging tasks previously shown to elicit robust DMN suppression. To reliably map suppression effects across the tasks, we implemented an equivalent rest-fixation “baseline” condition in each task from which to map relative suppression effects. Based on previous research, we hypothesized that we would observe a distinct suppression signature across the three tasks that encompassed traditionally recognized core DMN regions (namely, the MPFC) and would also extend to regions outside the network. Further, within this suppression signature, we hypothesized that the magnitude of suppression effects would be significantly correlated within individuals across tasks.

## Materials and methods

### Participants

One-hundred and four adolescents and young adults (15–25 years) were recruited to the study and completed the full imaging protocol. Participants were considered eligible if they were (i) without current or past diagnosis of a DSM-IV Axis I mental disorder as assessed by the Structured Clinical Interview (SCID; First et al. 1997); (ii) competent English speakers with an estimated IQ greater than 85 (Wechsler 2001); (iii) not taking any psychoactive medication; (iv) not pregnant; and (v) had no further contraindications to magnetic resonance imaging. The study was approved by the Melbourne Health Human Research Ethics Committee, Victoria, Australia. Participants (and their parents if aged under 18 years) provided informed consent prior to participation. A total of 19 participants were excluded due to: incidental findings (*n* = 4); acquisition failure (*n* = 9); excessive motion (*n* = 2); and poor behavioral performance defined as less than 80% accuracy in respective task conditions (*n* = 4). The final sample comprised 85 participants (47 females) aged 15–25 years (*M* = 20.0, *SD* = 2.8) who successfully completed all 3 tasks.

### Experimental tasks

Participants completed three distinct blocked-design tasks that have been widely used in functional magnetic resonance imaging (fMRI) studies. The tasks were modeled to allow us to examine task-induced DMN suppression effects from a rest-fixation baseline condition, as in Harrison et al. (2011), as well as different cognitive processes engaged by the tasks. The tasks were completed sequentially in a single scanning session. Brief task summaries are described below with further details provided in the Supplementary Material.

#### Cognitive reappraisal task

The cognitive reappraisal task examined emotion reactivity and regulation to negative stimuli and has been the focus of previous work by our group (Stephanou et al. 2016; Steward et al. 2021). Briefly, the task comprised three conditions: two passive “look” conditions (comprising blocks with either neutral or negative images) and an active “reappraise” condition (comprising only negative images). Participants were presented with images depicting social scenes and instructed to either “look” (i.e. attend to images without trying to alter their emotions) or “reappraise” (i.e. use cognitive reappraisal strategies—learned during a pre-task training session—to attenuate their emotional response to negative images). The full description of the reappraisal training can be found in the Supplementary Material. Each condition block was 30 s (2 s instruction, 6 s presentation of consecutive images x 4, and 4 s self-rating of negative affect where participants rated their emotion reactivity to the presented stimuli). There were 8 blocks per condition (3 x 8 = 24 task blocks) that were interleaved with 10 s rest-fixation periods (25 in total) where participants viewed a centrally presented crosshair. Time to complete the task was ~16 min (~12 min allocated to task-activity and ~4 min of rest-fixation).

#### Emotional face-matching task

The emotional face-matching task is a variation of the task described by Hariri et al. (2000), and assessed implicit emotional processing to negatively valanced facial stimuli. The task has been examined in previous work by our group (Jamieson et al. 2021). In brief, the task comprised three conditions: a lower-demand (relatively easy) “shape-matching” condition and two higher-demand (relatively more difficult) “face-matching” conditions (comprising blocks of either sad or fear faces). Participants were presented with triples of images of either circular shapes or faces (depicting sad or fearful expressions) and instructed to match the shape (shape-matching condition) or the gender of the face (face-matching condition) at the top of the screen to the corresponding shape or gender at the bottom left or right of the screen. Responses (accuracy and response time; RT) were recorded by pressing buttons 1 (for left) or 2 (for right) on a 4-button-box. Each condition block was 24 s (presentation of 6 consecutive images each for 3.75 s followed by 0.25 s pause). There were 6 blocks per condition (3 x 6 = 18) with sad and fear face blocks counterbalanced between participants. Blocks were interspersed with 10 s rest-fixation periods (19 in total) where participants fixated on a central crosshair. Task duration was ~10 min (~7 min of task-based activity and ~3 min of rest-fixation).

#### Self-referential processing task

The self-referential processing task assessed internally- and externally-directed attentional processes and has been the focus of previous work by our group (Davey et al. 2016). Briefly, the task comprised two conditions: an internally-directed “self-referential” condition and an externally-directed “letter-discrimination” condition. Participants were presented with words (i.e. trait adjectives) and asked to appraise whether a presented word described them (self-referential condition) or assess if the word had 4 or more vowels (letter-discrimination condition). Responses (selection/accuracy, respectively and RT) were recorded by pressing buttons 1 (yes) or 2 (no) on a 4-button-box. Each condition block was 32 s (2 s instruction and 6 words presented for 5 s each). There were 8 blocks per condition (2 x 8 = 16) interleaved with 10 s rest-fixation periods (17 in total) in which participants fixated on a central crosshair. Task duration was ~11 min (~8 min of task-based activity and ~3 min of rest-fixation).

### Image acquisition

A 3 T General Electric Signa Excite system with an 8-channel phased-array head coil was used in combination with ASSET parallel imaging. The functional sequences consisted of a single shot gradient-recalled echoplanar imaging sequence in the steady state (repetition time, 2,000 ms; echo time, 35 ms; and pulse angle, 90°) in a 23-cm field-of-view, with a 64 x 64-pixel matrix and a slice thickness of 3.5 mm (no gap). Thirty-six interleaved slices were acquired parallel to the anterior–posterior commissure line with a 20° anterior tilt to better cover ventral prefrontal brain regions. The total sequence durations were as follows: “cognitive reappraisal task,” 16 min and 10 s, corresponding to 485 whole-brain volumes; “emotional face-matching task,” 10 min and 22 s, corresponding to 311 whole-brain volumes; “self-referential processing task,” 11 min and 22 s, corresponding to 341 whole-brain echoplanar imaging volumes. For each sequence, the first 4 volumes from each run were automatically discarded to allow for signal equilibration. A T1-weighted high-resolution anatomic image was acquired for each participant to assist with functional time-series coregistration (140 contiguous slices; repetition time, 7.9 s; echo time, 3 s; flip angle, 13°; in a 25.6-cm field-of-view, with a 256 x 256-pixel matrix and a slice thickness of 1 mm). To assist with noise reduction and head immobility, all participants used earplugs and had their heads supported with foam-padding inserts. Each task was programmed and presented with Paradigm software (http://www.paradigmexperiments.com) and run on a Dell computer. The liquid-crystal display (LCD) screen used to present stimuli was visible via a reverse mirror mounted to the participants’ head coil. Behavioral responses were recorded using an optical-fiber 4-button-box that participants were familiarized with prior to scanning.

### Image preprocessing and first-level analysis

Imaging data were transferred and processed on a Unix-platform running MATLAB version 9.4 (The MathWorks Inc., Natick, MA, USA). Preprocessing was performed with Statistical Parametric Mapping software (SPM12; Wellcome Trust Centre for Neuroimaging, London, UK). Motion correction was performed by aligning each participants’ time-series to the first image using least-squares minimization and a 6-parameter (rigid body) spatial transformation. Motion fingerprint (Wilke 2012) was used to quantify scan-to-scan head motion. Participants were excluded if movement exceeded 3 mm mean total displacement (~1 native voxel). The realigned functional images were then coregistered to each participant’s respective T1 anatomical scan, which were segmented and spatially normalized to the International Consortium for Brain Mapping (ICBM152) template using the unified segmentation approach. These functional images were interpolated to 2 mm isotropic resolution and were smoothed with a 6 mm full-width-at-half-maximum Gaussian filter.

Each participants’ preprocessed time-series was then included in a first-level general linear model (GLM) analysis in SPM12. Each task was modeled separately with rest-fixation blocks forming the implicit baseline. Primary task regressors were created by specifying the onset and duration of each condition block with each task, followed by convolution with a canonical hemodynamic response function. For the cognitive reappraisal task, primary regressors included the look-neutral, look-negative and reappraise blocks with instruction and rating blocks entered as nuisance regressors. For the emotional face-matching task, primary regressors included the shape-matching and 2 gender-matching (sad and fear) faces blocks. For the self-referential processing task, primary regressors included the self-referential and letter-discrimination word blocks with the instruction block entered as a nuisance regressor. Maximum likelihood parameter estimates were calculated at each voxel using the GLM and an AR(1) model of serial autocorrelations. A high-pass filter (1/128 s) accounted for low-frequency noise, while temporal autocorrelations were estimated using a first-order autoregressive model. First-level (single-subject) SPM contrast images were estimated for the following primary contrasts of interest from each task: (i) rest > reappraise; (ii) rest > face-matching; and (iii) rest > letter-discrimination. These contrasts were the most appropriate for our current analysis examining the consistency of DMN suppression effects; allowing us to identify brain regions that showed relative suppression from rest during active externally oriented cognitive task performance.

### Mapping DMN suppression effects in each task

To address our primary aim of identifying brain regions showing the greatest task-induced suppression relative to rest, primary contrast images for each participant were entered into a second-level random-effects GLM using one-sample *t*-test designs. Group whole-brain statistical parametric maps were thresholded using a false-discovery rate error (FDR) correction *P* < 0.05, with a minimum cluster extent of 10 contiguous voxels.

### Identifying common DMN suppression effects across tasks via conjunction

To identify common areas of suppression apparent across the 3 task contrasts, a group-level conjunction analysis (Friston et al. 1999; Friston et al. 2005) was conducted using a one-way ANOVA testing the conjunction null hypothesis (*P*_FDR_ < 0.05; 10 voxel cluster extent threshold). The cluster map of common suppressed regions from this analysis was also to be used as an implicit mask in the subsequent correlation analysis (see below).

### Examining the consistency of DMN suppression within participants

To further address our primary aim and identify common and distinct regions of relative suppression within individuals across the tasks we computed spatial correlations between pairs of suppression maps using the Biological Parametric Mapping (BPM) toolbox (Casanova et al. 2007; https://www.nitrc.org/projects/rbpm). The BPM toolbox is fully implemented in SPM and uses the GLM theoretical framework with random-field theory allowing for statistical inference based on different imaging maps or modalities. Unlike a SPM GLM or conjunction analysis, BPM can estimate voxel-wise analyses between functional imaging data by constructing a design matrix and performing computations on a voxel-by-voxel basis to produce parameter estimates that are used to generate statistical maps (such as positive and negative correlation maps). For our purposes, it allowed us to estimate whole-brain voxel-wise comparisons of pairwise correlations between inter-dependent suppression cluster maps to identify overlap of positive cross-task correlations within individuals. To do this, each individuals’ first-level contrast images from each task were used to compute voxel-wise pairwise correlations between each task-pair (“rest > task”) contrasts: (i) “reappraise & face-matching”; (ii) “face-matching & letter-discrimination”; (iii) “letter-discrimination & reappraise.” This allowed us to determine the consistency of task-induced suppression within participants; namely, whether individuals who showed robust suppression in one task similarly showed robust suppression in another. The conjunction mask, described above, was used to constrain the correlation analyses to regions of common suppression across tasks. The SPM output maps were analyzed with a statistical threshold of *r* > 0.4 to identify moderate-to-strong positive associations. Each cross-task pairwise correlation output map was then estimated to visualize spatial overlap of common regions where the magnitude of suppression was correlated within individuals across all tasks.

## Results

### Behavioral task performance

Behavioral data for each task are presented in Table 1. Accuracy and RT scores are reported for the emotional face-matching task and the letter-discrimination condition of the self-referential processing task. As accuracy for the self-referential condition was not an appropriate behavioral measure, response rate (RR) to word stimuli is reported. Given the nature of the cognitive reappraisal task, neither accuracy nor RT were recorded. For this task, participant scores on the negative affect self-rating scale and RT for the “look” and “reappraise” conditions are reported as behavioral measures. RT scores for the rating of reappraise images, face-matching and letter-discrimination conditions were significantly slower compared to their respective counterpart conditions suggesting these task conditions were relatively more cognitively demanding (Table 1).

**Table 1:** Behavioral task performance

Task	Condition	Mean (SD)	Mean (SD)	* T*_RT_	*P*_RT_
		Negative affect rating	RT in seconds		
Cognitive reappraisal task	Look	1.8 (0.3)	1.20 (0.33)	−8.92	<0.001
	Reappraise	2.0 (0.5)	1.53 (0.49)		
		RR^†^ or Accuracy %	RT in seconds		
Emotional face-matching task	Shape-matching	97.8 (2.9)	0.76 (0.16)	−40.92	<0.001
	Face-matching	96.7 (2.0)	1.91 (0.33)		
Self-referential processing task	Self-referential	99.0^†^ (3.3)	1.66 (0.34)	−5.69	<0.001
	Letter-discrimination	96.5 (4.0)	1.94 (0.43)		

Note. Look condition = average of neutral and negative image conditions. Face-matching condition = average of sad and fear facial expression conditions. RT = response time (calculated from percentage correct for the face-matching and letter-discrimination conditions). RR^†^ = response rate (for the self-referential condition where participants were asked to appraise if the presented word described them). SD = standard deviation. *T_RT_* = *t*-statistic for RT comparison. *P*_RT_ = *P* value associated with RT comparison.

### DMN suppression effects in each task

**Fig. 1:**
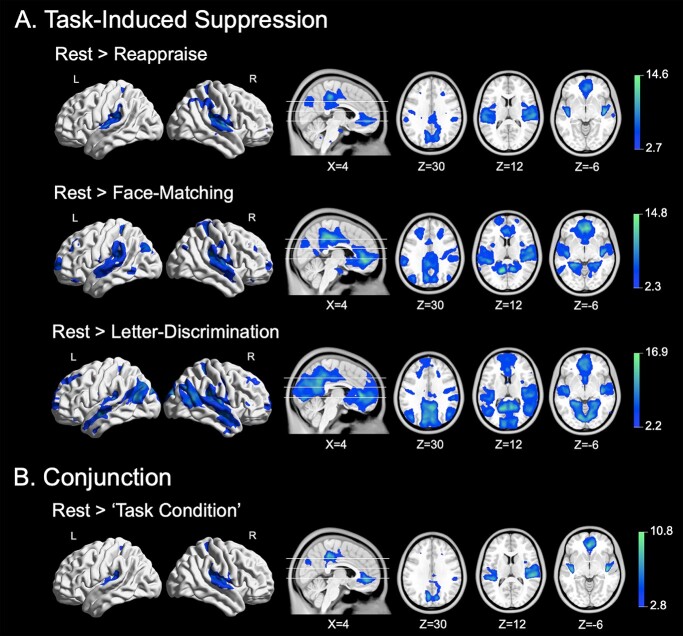
Significant whole-brain task-induced suppression and conjunction (rest > “task condition”) results. A) Significant task-induced suppression associated with each of the 3 tasks. B) Conjunction analysis showing anatomical overlap of task-induced suppression across the 3 tasks. Displayed contrast and conjunction maps are thresholded SPM *t*-statistic images (*P*_FDR_ < 0.05). Colorbar represents *t*-statistics.

The group-level GLM analysis was used to address our primary aim and identify common and distinct regions showing relative suppression from rest in each task. As seen in Fig. 1A, we observed a general pattern of suppression in each task that broadly encompassed major DMN regions, including the posterior and anterior midline cortices, and lateral parietal cortices. Notably, suppression also extended to other cortical areas, including a broad area of the posteromedial cortex, and medial parietal areas encompassing the posterior and middle insular and surrounding cortex. The corresponding anatomical coordinates of all suppressed regions and their associated statistical magnitudes and extents for each task are provided in Supplementary Table S3. A map of distinct task-induced suppression associated with each task is provided in Supplementary Fig. S4. Supplementary Fig. S5 presents group-level (model predicted) time-series responses for the common peak clusters of suppression for each task. The results of this supplementary time-series analysis endorse the consistency of the task evoked neural dynamics as highlighted by the GLM findings. Further information of the activity time-series analysis is detailed in the Supplementary Material.

### Common DMN suppression across tasks

To confirm the observation of common anatomical overlap of suppressed regions in each task, we performed a group-level conjunction analysis of the 3 independent second-level cluster maps. As seen in Fig. 1B, the conjunction analysis revealed a robust and consistent pattern of common DMN suppression across the three tasks that broadly encompassed traditionally recognized DMN regions but also extended to adjacent regions that have previously been shown to demonstrate distinct suppression effects. Regions of common suppression included the left rostral ACC extending to the subgenual ACC and frontal pole; the dorsal PCC extending to mid-cingulate cortex; a distinct cluster in the precuneus extending to cuneus; the ventral posterior insula cortex extending to parietal operculum/second somatosensory cortex; the dorsal premotor cortex; right posterior primary motor cortex; and right dorsal mid-insula cortex. The anatomical coordinates of all clusters and their associated statistical magnitudes and extents are provided in Table 2.

**Table 2:** Conjunction of task-induced suppression

	Coordinates					
Brain Region	*x*	*y*	*z*	Cluster size	Peak *t*	*Z*
Posterior insular cortex (R)	40	−16	2	3,849	10.80	>10
Posterior cingulate cortex (R)	10	−32	42	4,651	9.82	>9
Posterior insular cortex (L)	−40	−18	−2	2,549	9.64	>9
Anterior cingulate cortex (R)	0	42	−6	1,669	9.52	>9
Mid insular cortex (R)	34	4	10	42	5.31	5.17
Middle frontal gyrus (R)	28	30	34	66	3.96	3.89
Post-central gyrus (R)	40	−16	38	82	3.94	3.88
Pre-central gyrus (R)	22	−16	60	62	3.70	3.65
Pre-central gyrus (L)	−18	−24	60	34	3.64	3.59
Middle frontal gyrus (L)	−28	30	34	21	3.19	3.15

Note: Coordinates and statistics for the conjunction analysis of task-induced suppression across the 3 task contrasts: rest > reappraise; rest > face-matching; rest > letter-discrimination. Coordinates (peak voxel) in MNI space (mm). Cluster size = # of voxels (> 10 continuous). Magnitude and extent statistics correspond to a minimum threshold of *P*_FDR_ < 0.05. Peak T & Z = SPM T-score and Z-score statistics. L = left hemisphere; R = right hemisphere.

### Consistency of the magnitude of DMN suppression within participants

**Fig. 2:**
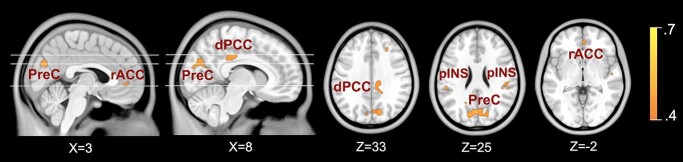
Spatial overlap of the 3 cross-task pairwise BPM correlations: “rest > reappraise & rest > face-matching”; “rest > face-matching & rest > letter-discrimination”; “rest > letter-discrimination & rest > reappraise.” Displayed maps show the spatial (i.e. regional) overlap of the 3 correlation output maps that were analyzed with a statistical threshold of *r* > 0.4. Overlap maps were generated by creating a conjunction map of the 3 pairwise correlation maps. PreC = precuneus. dPCC = dorsal posterior cingulate cortex. rACC = rostral anterior cingulate cortex. pINS = posterior insula. Colorbar represents average correlation statistics.

Figure 2 shows the spatial overlap of cross-task pairwise correlations in commonly suppressed DMN regions. Across the 3 tasks, we observed significant within-participants correlations in the magnitude of DMN suppression in the IPL and precuneus, dorsal PCC extending to the mid-cingulate cortex, MPFC/rostral ACC, posterior insula (pINS), and frontal eyes fields (FEF). The specific anatomical coordinates of the spatially overlapping pairwise correlation clusters and their associated statistical magnitudes and extents are provided in Table 3. Individual maps of cross-task pairwise correlations are provided in Supplementary Fig. S6. Pairwise Pearson’s correlations are provided in Supplementary Table S4. Anatomical coordinates and statistical magnitudes and extents for the individual cross-task pairwise correlations are reported in Supplementary Table S5.

**Table 3:** Spatial overlap of cross-task pairwise correlations

	Coordinates				
Brain region	*x*	*y*	*z*	Cluster size	Peak *R*_avg_
Precuneus (L)	−4	−74	26	428	0.67
Supramarginal gyrus (R)	52	−24	28	36	0.61
Middle frontal gyrus (R)	26	34	32	11	0.60
Posterior insula cortex (R)	48	−20	4	95	0.58
Posterior cingulate cortex (R)	10	−28	34	85	0.57
Insular cortex (R)	52	−14	20	10	0.56
Anterior cingulate cortex (R)	2	48	0	12	0.53
Post-central gyrus (R)	64	−18	18	11	0.53
Superior temporal gyrus (L)	−62	−30	12	13	0.52
Inferior temporal gyrus (L)	−56	−36	24	22	0.51

Note: Coordinates and statistics for the spatial (i.e. regional) overlap of BPM pairwise correlations: “rest > reappraise & rest > face-matching”; “rest > face-matching & rest > letter-discrimination”; “rest > letter-discrimination & rest > reappraise.” Coordinates (peak voxel) are in MNI space (mm). Cluster size = # of voxels (>10 continuous). Magnitude and extent statistics correspond to a statistical threshold of *r* > 0.4. Peak *R*_avg_ = average correlation coefficient of the 3 cross-task pairwise correlations. L = left hemisphere; R = right hemisphere.

### Correlations with demographic and psychological measures

As a secondary aim, we examined associations between the strength of overall suppression with age, IQ (raw Weschler Test of Adult Reading [WTAR]; Wechsler 2001), and estimated age-adjusted Full-Scale IQ [FSIQ] scores), and a measure of ruminative style—the Ruminative Responses Scale (RRS; Treynor et al. 2003)—as rumination has frequently been implicated as modulating DMN activity (Hamilton et al. 2011). A suppression index was calculated by averaging the peak suppression values from the most robust clusters identified from the conjunction analysis (i.e. pINS, PCC, and rostral ACC). One participants’ suppression index was observed to be abnormally high (i.e. greater than 3 standard deviations from the group average) and was removed from the subsequent correlation analyses. There were no significant associations between the suppression index and age (*r* = −0.15, *P* = 0.164), IQ_WTAR_ (*r* = −0.18, *P* = 0.104), IQ_FSIQ_ (*r* = −0.19, *P* = 0.083), nor RRS score (*r* = 0.02, *P* = 0.874). Descriptive statistics for these variables are reported in Supplementary Table S6.

## Discussion

In this study, we investigated task-induced modulation of DMN suppression across multiple externally directed active cognitive tasks performed by the same participants. From the broader patterns of DMN suppression in response to each task, we identified a distinct “subnetwork” across the tasks that showed strong anatomical (i.e. spatial) and functional (i.e. magnitude) consistency (Fig. 1A and B). Specifically, the three tasks evoked common suppression in the MPFC, posteromedial cortex, middle frontal gyrus (MFG), FEF, pINS, and surrounding cortex. These regions generally corresponded with those reported in previous work investigating task-induced suppression at higher levels of task load (McKiernan et al. 2003; Tomasi et al. 2006; Mayer et al. 2010; Harrison et al. 2011; Davey et al. 2016). Further, we found that the magnitude of suppression within each region comprising this subnetwork was significantly correlated within individuals across the tasks (Fig. 2). Together, the current findings suggest this distinct suppression signature may be a domain-general mechanism that represents a stable feature of human brain function important for optimizing external goal-directed performance.

Recent conceptualizations of the DMN suggest it consists of core regions (i.e. MPFC, PCC, and IPL) with adjacent regions recruited in response to specific task demands (Buckner et al. 2008; Andrews-Hanna et al. 2010; Buckner and DiNicola 2019). Moreover, a notable functional distinction has been identified in the PCC where the ventral subregion is more directly linked with the DMN and the dorsal subregion serves as a “hub” between the DMN and cognitive control networks (Leech et al. 2011; Leech et al. 2012; Leech and Sharp 2014). Therefore, while the MPFC represents a core component of the DMN, the other regions identified in our analysis extend beyond the core network. Our findings demonstrate that task-induced suppression during externally directed active task performance is only partly expressed in the core DMN and support previous findings (e.g. Harrison et al. 2011) that suggest suppression of additional regions become important under changing levels of task demand.

Distinct from the other core DMN regions, suppression of the MPFC has been consistently observed in most cognitive tasks (Shulman et al. 1997; Binder et al. 1999; Mazoyer et al. 2001) and levels of difficulty (McKiernan et al. 2003; McKiernan et al. 2006; Mayer et al. 2010; Harrison et al. 2011). Notably, the MPFC has been particularly linked to self-appraisal processes (Gusnard et al. 2001; Szpunar et al. 2007; Delahoy et al. 2022; Steward et al. 2022); a notion supported by our exploratory time-series analysis that showed sustained activity in the MPFC during the self-referential condition and sustained suppression during the letter-discrimination condition of the self-referential processing task (Supplementary Fig. S5C). Therefore, suppression of this region during active task engagement may reflect “true” disengagement of broad experiential self-related operations (Davey et al. 2016).

Extending this view, suppression of the pINS and FEF are often observed when attentional allocation to the external environment requires intense concentration and narrow visual focus (Tomasi et al. 2006; Mayer et al. 2010; Harrison et al. 2011; Davey et al. 2016). The pINS is associated with somatic sensory information processing relating to body-related afferent processes, feeling states, and interoception (Craig 2003; Craig 2009), while the FEF are involved in maintaining attention to peripheral locations (Kelley et al. 2008). Co-suppression of these regions may reflect inhibition of broader aspects of conscious self-awareness of internal processes as a person becomes progressively immersed in a task. Therefore, this domain-general suppression mechanism may act to minimize potentially distracting processes that may interfere with task performance—underlying the concept of “losing oneself” in a task.

While the dominant view of DMN function is supporting internally focused processes, converging evidence is elucidating its role in externally directed cognition, including facilitating improved task performance (Hahn et al. 2007). Recent studies have observed increased DMN activity during large attentional shifts such as switching between demanding task epochs and low demand or resting conditions (Crittenden et al. 2015; Smith et al. 2018). This work suggests that the network may play an important role in facilitating cognitive transitions—a core feature of cognitive control that is critical for efficient goal-directed behavior. These findings support previous notions of the DMN as maintaining broad vigilance of environment cues to more readily respond to changing contexts (Shulman et al. 1997; Buckner et al. 2008). From this perspective, the DMN may be suppressed when cognitive operations are mostly similar, but as changes in cognitive context become sufficiently large (i.e. dissimilar) or triggered by sudden shifts in visuospatial attention, suppression may shift to activation to facilitate adaptive reconfiguration to new contexts.

Moreover, sustained activity may also explain why high metabolism is often observed in DMN regions during tasks. In an important recent study, Stiernman et al. (2021) found neural activity (i.e. glucose metabolism) in core DMN regions remained high or increased during task conditions despite relative decreases in blood-oxygen-level-dependent (BOLD) response. They interpreted high metabolic activity as reflecting ongoing regulation of brain dynamics during cognitive operations, such as shifting between rest and task states, which may not be fully captured by signal averaging with fMRI. Notably, the only area to show concomitant decreases was a small cluster in the anterior MPFC. The findings presented by Stiernman et al. provide robust support for functional heterogeneity within the DMN and propose an active suppression account of some regions within the network. Considered together, these findings may explain why, given the interspersed rest versus relatively demanding or dissimilar task condition in our analysis, we observed common suppression only in the MPFC/rostral ACC. This further reinforces the notion that the MFPC may represent a true or core “default” region that reflects processes relating to the “self” that are first to disengage when attending to external stimuli (Davey and Harrison 2022). This is also reflected in our exploratory time-series analysis that revealed the MPFC was the only region to distinctly show immediate and sustained suppression to task conditions, consistent with the traditional viewpoint of DMN activity (Supplementary Fig. S5).

The novelty of the present work is that we found a subnetwork of task-induced suppressed regions that were not only common across the three tasks tapping different cognitive functions, but that the magnitude of suppression in these regions were significantly correlated within individuals across tasks. This suggests that, despite some heterogeneity across tasks, there may exist a stable domain-general suppression mechanism in the DMN that potentially spans across various contexts where suppression of DMN activity is required for optimizing goal-directed behavior.

Although a key strength of this study was investigating task-induced suppression across three distinct cognitive tasks in the same sample, a few limitations should be noted. First, we did not assess modulated levels of task difficulty within each task as our primary goal was to investigate the consistency of task-induced suppression during externally directed cognitive task performance across different attentionally demanding tasks. Second, previous work has shown age-related differences during cognitive tasks, with younger adults generally showing better efficiency at suppressing the DMN in response to cognitive demand compared with older adults (Persson et al. 2007; Park 2010; Spreng et al. 2010). Thus, the age of our cohort may limit the generalizability of our findings beyond young adult populations.

## Conclusion

Our findings demonstrate that task-induced suppression during externally directed cognitive tasks evokes a distinct suppression subnetwork that is partially expressed in the core DMN but also extends to regions involved in broader aspects of self-awareness and cognitive control. Further, we found that the magnitude of suppression in these regions were significantly correlated within individuals across tasks suggesting this may be a domain-general mechanism that is a stable, task-independent feature of cognitive function that optimizes external goal-directed behavior. The current study contributes key novel insights into the anatomical and functional consistency of DMN suppression during active engagement of cognitive tasks and its diverse contributions to efficient cognition function.

## Supplementary Material

CerCor-2022-00334_CL_SupplementaryMaterial_FINAL_bhac361
