# Supplementary material for: A distinct intra-individual suppression subnetwork in the brain’s default mode network across cognitive tasks
Source: Cereb Cortex. 2022 Sep 20;33(8):4553–61. doi: 10.1093/cercor/bhac361 (PMC10110429; doi:10.1093/cercor/bhac361)
Supplement: CerCor-2022-00334_CL_SupplementaryMaterial_FINAL_bhac361 [file cercor-2022-00334_cl_supplementarymaterial_final_bhac361.docx]

**Supplementary Material

A distinct intra-individual suppression subnetwork in the brain’s default mode network during cognitive tasks**

Christine A. Leonards, Ben J. Harrison, Alec J. Jamieson, Trevor Steward, Silke Lux, Alexandra Philipsen, Christopher G. Davey

**Contents**

**Supplementary Methods**

Cognitive Reappraisal Task 2

Cognitive reappraisal task full description 2

Figure S1. Cognitive reappraisal task design 3

Cognitive reappraisal (pre-task) training 3

Cognitive reappraisal task picture stimuli 4

Table S1. Cognitive reappraisal task picture stimuli list 5

Emotional Face-Matching Task 7

Emotional face-matching task full description 7

Figure S2. Emotional face-matching task design 8

Emotional face-matching task picture stimuli 8

Self-Referential Processing Task 9

Self-referential processing task full description 9

Figure S3. Self-referential processing task design 10

Table S2. Self-referential processing task word stimuli list 10

**Supplementary Results**

Table S3. Significant task-induced suppression associated with each task 11

Figure S4. Distinct task-induced suppression associated with each task 12

Regional dynamic activity time-series analysis 13

Figure S5. Regional dynamic activity time-series for each task 13

Figure S6. Cross-task pairwise correlations of task-induced suppression 15

Table S4. Cross-task pairwise Pearson’s correlations of task-induced suppression 16

Table S5. Cross-task pairwise BPM correlations for each task-pair 17

Table S6. Descriptive statistics for the suppression index, demographic and psychological variables 18

**Supplementary References**

Reference list 19

**Supplementary Methods**

**Cognitive Reappraisal Task**

***Cognitive Reappraisal Task Full Description***

The developed block-design cognitive reappraisal task followed general features of common emotion regulation paradigms (Phan et al., 2005) and was designed specifically to assess emotion reactivity and regulation to negative social stimuli using cognitive regulation strategies (Stephanou et al., 2016). Consistent with prior studies the task involved three conditions – ‘Look-Neutral’, ‘Look-Negative’, and ‘Reappraise’ – presented in an ABC design with 8 blocks per condition (i.e., 8 x 3 = 24 blocks in total). See Figure S1. Each task block was 30 s. At the beginning of each block, participants were presented with a word for 2 s instructing them to either “Look” (i.e., attend to neutral or negative images without trying to alter their emotions elicited by the image) or “reappraise” (i.e., use reappraisal strategies – learned during a pre-task training session prior to scanning – to attenuate their emotional response to negative images). Further information regarding the training session is below. All blocks consisted of the presentation of 4 consecutive neutral or negative images (each for 6 s) depicting complex social scenes taken from the International Affective Picture System (IAPS; Lang et al., 2008), the Empathy Picture System (EPS; Geday et al., 2003) databases and online sources. Task stimuli is described below (task picture stimuli list is presented in Table S1). Immediately following the presentation of stimuli in each task block, participants were prompted to rate their negative affect in response to viewing the 4 consecutive images (cue: “How bad do you feel?)”. Responses were recorded by pressing buttons 1 to 4 (1 = not bad at all; 4 = very bad) with the dominant hand on an MRI-compatible optical-fiber 4-button-box that they were familiarized with prior to scanning. The task blocks were interspersed with 10 s rest periods in which participants viewed a fixation crosshair presented in the center of the screen. The task ended with an additional 10 s rest period. Time to complete the task was ~16 mins (12 mins allocated to task-activity and 4 mins and 10 secs of rest-fixation). For the purposes of our current analysis examining DMN suppression effects, our primary contrast of interest was the direct comparison of rest versus the ‘reappraise’ condition (rest > reappraise) as this “active” condition captured the suppression of activity that occurs in the default mode network, relative to spontaneous rest, due to increased cognitive demand required for effortful reappraisal of images.

**Figure S1**

*Cognitive Reappraisal Task Design*


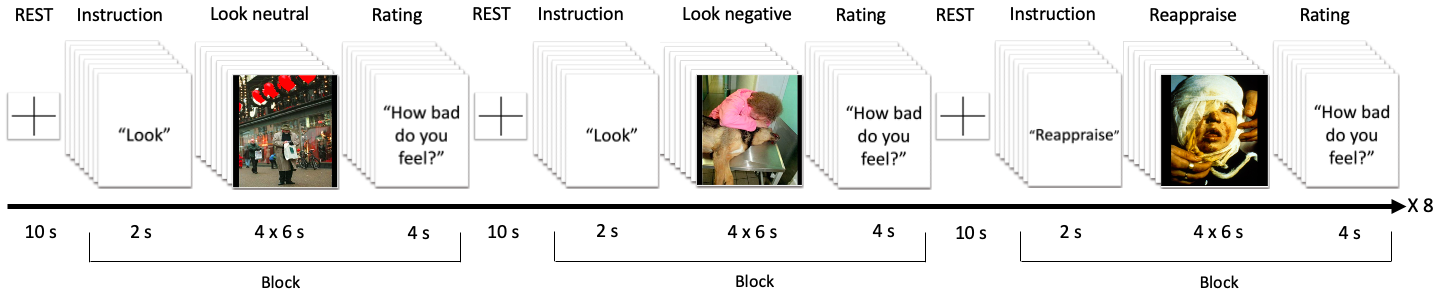


*Note.* Design of the cognitive reappraisal paradigm. Participants were presented with neutral or negative images in an ABC block design and instructed to either “look” (i.e., attend to neutral or negative images without altering their emotional response) or “reappraise” (i.e., use pre-learned cognitive reappraisal strategies to reduce their emotional response to negative images). Task blocks were interspersed with rest conditions.

***Cognitive Reappraisal (pre-task) Training***

Participants were familiarized with cognitive reappraisal strategies in the hour prior to scanning using a training protocol adapted from work by McRae et al. (2012).

Participants were presented with several practice images (that did not appear in the experiment) and prompted to demonstrate reappraisal by narrating aloud their re-interpretation of each image based on three types of re-interpretations that were suggested: (i) it is not real (e.g., it is just a scene from a movie); (ii) things will improve with time (e.g. whatever is going wrong will resolve over time); and (iii) things are not as bad as they appear to be (e.g., the situation looks worse than it is; it could be a lot worse; or at least it is not me in that situation). If a participants’ responses indicated that they were using a non-cognitive strategy (e.g., looking away or attending to non-emotional aspects of the picture), the participant was redirected to the three example strategies mentioned above. When the experimenter determined from a participant’s narration that they could utilize appropriate cognitive reappraisal strategies and within the desired time (i.e., 6 s per image), the participant completed several practice blocks of the experimental task independently in preparation for the scan. Although not the focus for this study, compliance with reappraisal strategies during the scan was assessed by a brief post-scan questionnaire that was designed to evaluate both participants’ perceived frequency of reappraisal strategy use as well as their use of avoidance or non-cognitive strategies (i.e., only looking at the non-emotional aspects of the picture, looking away or closing their eyes), which participants rated on a scale from 1 to 5 (1 = Never; 5 = Always).

***Cognitive Reappraisal Task Picture Stimuli***

The task was presented with Paradigm software (https://www.paradigmexperiments.com) on a Dell computer. The LCD screen that presented stimuli was visible via a reverse mirror mounted to the participants’ head coil. Images depicted complex social scenes that were taken from the International Affective Picture System (IAPS; Lang et al., 2008), the Empathy Picture System (EPS; Geday et al., 2003) databases and online sources. Images from the IAPS database normative ratings were used to guide the image selection. Specifically, images were considered for inclusion in the neutral subset if they have a valence rating between 4 and 6, and an arousal rating ≤ 2 (i.e., they were neutral in valence and weak in arousal). In contrast, images were considered for inclusion in the negative subset if their normative valence was ≤ 3.5 and arousal rating was ≥ 4, (i.e., they were negative in valence and moderate-to-high in arousal). Images from the EPS were categorized by valence on a 7-point scale (-3 = most discomfort; 3 = most comfort), and according to this scale images are pre-classified as negative, neutral, and positive. Images were classified as negative if all raters scored them ≤ -1; neutral if all raters scored them as between -1 and 1; and positive if all raters scored them ≥ 1. Images from the EPS database were considered for inclusion in this study if they were categorized according to these criteria as negative or neutral. To ensure comparability of pictures selected from different sources, individual images were rated for valence and arousal using standardized 9-point Self-Assessment Manikin Scale (1 = most unpleasant/least arousing; 9 = most unpleasant/most arousing). Picture stimuli (both negative and neutral pictures) were also selected to match for general content (including number of faces and figures) and differences in luminance and complexity were kept minimal. In the end, a total 32 neutral and 64 negative pictures were selected for use (see Table S1). Negative picture stimuli were divided into two picture-sets – matched for valence and arousal (t-tests, *p* = 0.23) ­– that were assigned to the aversive (i.e., ‘Look-Negative’ and ‘Reappraise’) stimulus conditions with conditions counterbalanced across participants.

**Table S1**

*Cognitive Reappraisal Task Picture Stimuli List*

| Source | Description | Number |
| --- | --- | --- |
| *Neutral Images* | | |
| IAPS | Mother/Child | 2359 |
| IAPS | GirlMakeup | 2308 |
| IAPS | LonelyBoy | 2272 |
| IAPS | Factoryworker | 2393 |
| IAPS | NeutralMale | 2493 |
| IAPS | NeutralFemale | 2440 |
| IAPS | Makeup | 2032 |
| IAPS | Smoking | 2749 |
| IAPS | Shopping | 2745.1 |
| IAPS | Female | 2400 |
| IAPS | Couple | 2396 |
| IAPS | Female | 2026 |
| EPS | Student/Classroom | Set 2_28_Neutral Situations |
| EPS | Labworkers | Set 2_24_Neutral Situations |
| EPS | NeutralMales | Set 1_21_Neutral Situations |
| EPS | Females/BusyStreet | Set 1_17_Neutral Situations |
| EPS | MaleChefs | Set 1_16_Neutral Situations |
| EPS | StatementReading | Set 1_15_Neutral Situations |
| EPS | DiplomatsMeeting | Set 1_9_Neutral Situations |
| EPS | Voting | Set 2_9_Neutral Situations |
| EPS | MaleShopping | Set 1_8_Neutral Situations |
| EPS | FemaleShopping | Set 2_6_Neutral Situations |
| EPS | NeutralMale | Set 1_3_Neutral Situations |
| EPS | Shopping | Set 2_2_Neutral Situations |
| EPS | ManShopping | Set 2_1_Neutral Situations |
| EPS | FactoryWork | Set 1_1_Neutral Situations |
| EPS | Students/Classroom | Set 1_7_Neutral Faces |
| EPS | NeutralFemale | Set 1_5_Neutral Faces |
| EPS | ManShopping | Set 2_4_Neutral Situations |
| EPS | SportCommentor | Set 1_4_Neutral Situations |
| EPS | NeutralMale | Set 1_23_Neutral Situations |
| EPS | NeutralMale | Set 1_14_Neutral Situations |
| *Negative Images A* | | |
| IAPS | Fire | 9921 |
| IAPS | Assault | 9429 |
| IAPS | Assault | 9428 |
| IAPS | Soldier | 9421 |
| IAPS | Assault | 9419 |
| IAPS | Execution | 9414 |
| IAPS | Soldier | 9410 |
| IAPS | CryingFemale | 9332 |
| IAPS | Soldiers | 9163 |
| IAPS | Police | 6834 |
| IAPS | Attack | 6563 |
| IAPS | InjuredChild | 3301 |
| IAPS | Hospital | 3220 |
| IAPS | GrievingFemale | 2141 |
| IAPS | Toddler | 2095 |
| EPS | DeadGirl | Set 2_9_Unpleasant Situations |
| EPS | Skeletons | Set 1_9_Unpleasant Situations |
| EPS | ChildrenCoffins | Set 2_4_Unpleasant Situations |
| EPS | DistressedMale | Set 1_30_Unpleasant Situations |
| EPS | Funeral | Set_2_3_Unpleasant Situations |
| EPS | Assault | Set_1_29_Unpleasant Situations |
| EPS | GrievingFemale | Set_1_26_Unpleasant Situations |
| EPS | DeadMale | Set_1_24_Unpleasant Situations |
| EPS | DeadBodies | Set_2_17_Unpleasant Situations |
| EPS | SadChild | Set_1_14_Unpleasant Faces |
| EPS | GrievingFemale | Set_1_12_Unpleasant Faces |
| EPS | DeadBodies | Set_2_25_Unpleasant Situations |
| EPS | BurntMale | Set_2_29_Unpleasant Situations |
| Online | StarvingChild | http://reapimage.blogspot.com.au/ |
| Online | DomViolence | http://reapimage.blogspot.com.au/ |
| Online | BatteredFemale | http://reapimage.blogspot.com.au/ |
| Online | CarCrash | http://reapimage.blogspot.com.au/ |
| *Negative Images B* | | |
| IAPS | Burial | 9430 |
| IAPS | DeadMale | 9412 |
| IAPS | WarVictim | 9250 |
| IAPS | StarvingChild | 9075 |
| IAPS | StarvingChild | 9040 |
| IAPS | Police | 6838 |
| IAPS | Police | 6831 |
| IAPS | Attack | 6520 |
| IAPS | Soldier | 6212 |
| IAPS | DyingMale | 3230 |
| IAPS | BatteredFemale | 3181 |
| IAPS | OpenGrave | 3005.1 |
| IAPS | Funeral | 2799 |
| IAPS | DrugAddict | 2710 |
| IAPS | SadChildren | 2703 |
| IAPS | Hospital | 2205 |
| EPS | Deadfemale | Set_2_7_Unpleasant Situations |
| EPS | Deadbodies | Set_2_27_Unpleasant Situations |
| EPS | BusCrash | Set_1_25_Unpleasant Situations |
| EPS | Funeral | Set_1_23_Unpleasant Faces |
| EPS | DeadFarmer | Set_2_21_Unpleasant Situations |
| EPS | InjuredChild | Set_1_20_Unpleasant Situations |
| EPS | GrievingFemale | Set_2_19_Unpleasant Situations |
| EPS | CarCrash | Set_2_18_Unpleasant Situations |
| EPS | Soldier | Set_2_14_Unpleasant Situations |
| EPS | SadChild | Set_2_13_Unpleasant Situations |
| EPS | Hangedman | Set_1_1_Unpleasant Situations |
| EPS | StarvingChild | Set_1_16_Unpleasant Faces |
| EPS | DeadBodies | Set_2_23_Unpleasant Situations |
| Online | Execution | http://reapimage.blogspot.com.au/ |
| Online | BatteredFemale | http://reapimage.blogspot.com.au/ |
| Online | SadChild | http://reapimage.blogspot.com.au/ |

*Note.* The list of neutral and negative images are drawn from International Affective Picture System (IAPS; Lang et al., 2008), the Empathy Picture System (EPS; Geday et al., 2003) and online sources. Images were rated and matched for general content, valence and arousal.

**Emotional Face-Matching Task**

***Emotional Face-Matching Task Full Description***

The block-design emotional face-matching task assessed inhibitory processes to negatively-valanced facial stimuli and is a variation of the face matching task described by (Hariri et al., 2000). It comprised three conditions: a shape matching condition and two face matching conditions (comprising either sad or fearful facial expressions) presented in an ABC design with 6 blocks per condition (i.e., 6 x 3 = 18 blocks in total). The task was programmed in Paradigm software (https://www.paradigmexperiments.com) on a Dell computer. The LCD screen that presented stimuli was visible via a reverse mirror mounted to the participants’ head coil. Participants were shown three images – circular shapes or faces conveying emotional expressions that were sourced from the Radboud Faces Database (Langner et al., 2010) to convey the same facial expressions – one presented at the top center of the screen and two in the bottom left and right corners of the screen. In the shape matching conditions, participants were instructed to match the orientation of the circular shape presented at the top to the corresponding shape presented at the bottom; and it the face-matching conditions, participants were instructed to match the gender of the (sad or fearful) face at the top to the corresponding gender presented at the bottom of the screen (see Figure S2). Matching the gender rather than the emotional expression was chosen as the main component of the task as this better captures neural processes associated with cognitive demand. Order of block presentation for the face conditions was counterbalanced between participants (e.g., (A) Shapes, Sad, Fear; (B) Shapes, Fear, Sad). Participant responses (accuracy and response time) were recorded by pressing buttons 1 (left) or 2 (right) with the dominant hand on an MRI-compatible optical-fiber 4-button-box that participants were familiarized with prior to scanning. Duration of each block was 4 seconds (3.75 s followed by .25 s pause), totaling 24 s in total. The task blocks were interleaved with a 10 s rest-fixation period where participants fixated on a crosshair. An additional 10 s rest period was included at the end. Total time to complete the task was ~10 mins (comprising 7 mins and 20 s task-based activity and 3 mins and 10 s of rest-fixation). Our contrast of interest was rest > face-matching as this captured the relative suppression of activity that occurs in the DMN elicited during active engagement in the gender-matching task which was further intensified by the implicit interference of negative affect in the emotional facial expressions.

**Figure S2**

*Emotional Face-Matching Task Design*


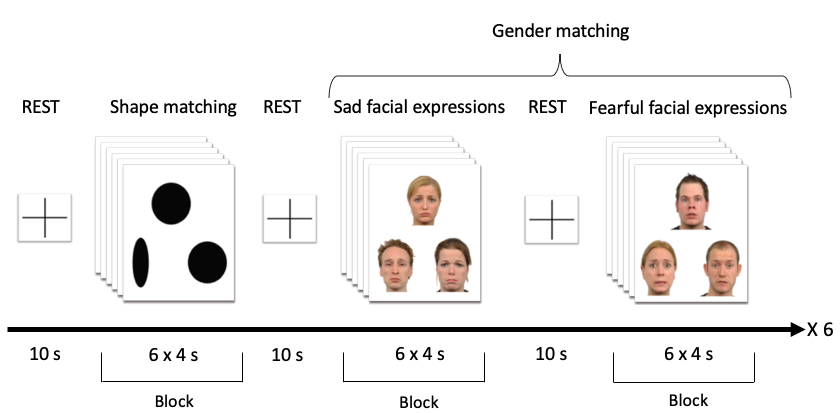


*Note*. Design of the face-matching paradigm. Participants were presented with triples of shapes or emotional faces (sad or fearful expressions) in an ABC block design. The task involved matching the image (shape or face) presented at top of the screen with the corresponding image presented at the bottom based on either the orientation of the shape (shape-matching condition) or gender of the face (face-matching condition). The face-matching condition was counterbalanced between participants (e.g., A [Shapes > SadFaces > FearFaces] and B [Shape > FearFaces > SadFaces]. Task blocks were interleaved with rest conditions.

***Emotional Face-Matching Task Picture Stimuli***

All of the face stimuli were collected from the Radboud Face Database (Langner et al., 2010). In total, 18 male and 18 female faces depicting sad and fearful expressions with a frontal gaze were used.

**Self-Referential Processing Task**

***Self-Referential Processing Task Full Description***

The block-design self-referential processing task was designed to emulate common features that were similar to previously published papers (e.g., Kelley et al., 2002; Heatherton et al., 2006; Moran et al., 2006; Whitfield-Gabrieli et al., 2011). The task assessed internally-directed self-appraisal processes and externally-directed attentional processing via two conditions: a ‘self-referential’ condition and a ‘letter-discrimination’ condition. Participants were presented with a word (i.e., trait adjective) on the center of the screen and asked either “Does this word describe you?” (self-referential condition) or “Does this word have 4 or more vowels?” (letter-discrimination condition). Task design is presented in Figure S3. Words were drawn from a frequently used lists of personality trait objectives (Anderson, 1968); specifically, from the subset of words rated as most ‘meaningful’. From those, 96 were selected based on their distribution around the median rating for ‘likeableness’ reported in the original dataset. Words with relatively neutral valence such as “skeptical”, “perfectionistic”, and “lucky” were selected and used in the task (see Table S2). These words were intentionally chosen to heighten self-appraisal but not conflate self-referential processing during the letter-discrimination condition. The two lists of 48 words that formed the self-referential and letter-discrimination conditions were matched on likeableness ratings and number of vowels. At the beginning of each block, participants were presented with the instructional question “Does this word describe you?” (self-appraisal condition) or “Does this word have four or more vowels?” (letter-discrimination condition) for 2 s in the center of the screen followed by the presentation of six consecutive trait words (5 s each; see Figure S3). Participants viewed 8 blocks per condition (2 x 8 = 16) which were counterbalanced across participants. Participants responded by pressing buttons 1 (yes) or 2 (no) on the optical-fiber 4-button-box. Each block-run was interleaved with a 10 s rest-fixation period where participants viewed a fixation cross. This task also ended with an additional 10 s rest period. Total time to complete the task was ~11 mins (8 mins and 32 secs allocated to task-activity and 2 mins and 50 secs of rest-fixation). For the purposes of our current analysis examining DMN suppression effects, our primary contrast of interest was the direct comparison of rest > letter-discrimination as this condition was designed to minimize the likelihood of off-task self-reflective processing and tap into externally-oriented attentional demand that triggers activity suppression of the default mode network.

**Figure S3**

*Self-Referential Processing Task Design*


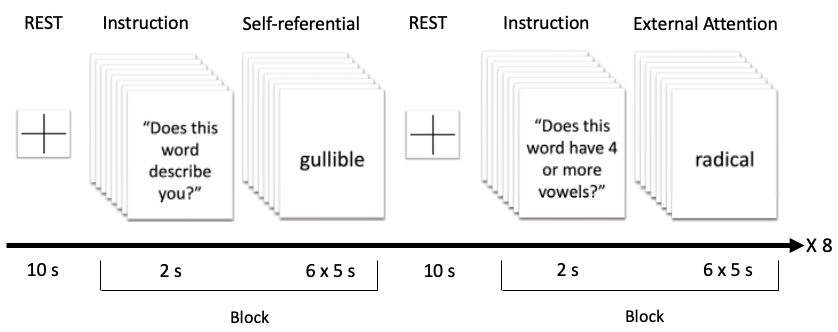


*Note.* Design of the self-referential paradigm. Participants were presented with words in an AB block design and asked whether the word described them (self-referential condition) or if the word had 4 or more vowels (letter-discrimination condition). A and B word lists were counterbalanced between participants. Task blocks were interspersed with rest conditions.

**Table S2**

*Self-Referential Task Word Stimuli List*

| List A |  | List B |  |
| --- | --- | --- | --- |
| inexperienced | bold | excitable | troubled |
| gullible | conventional | painstaking | eccentric |
| radical | wordy | silly | nonchalant |
| tough | passive | proud | unsophisticated |
| moderate | self-concerned | lucky | conservative |
| innocent | inhibited | opinionated | irreligious |
| unlucky | methodological | self-satisfied | overcautious |
| quiet | daring | mathematical | temperamental |
| satirical | theatrical | materialistic | daydreamer |
| fearless | unmethodical | solemn | impressionable |
| unpopular | rebellious | hesitant | fearful |
| extravagant | self-contended | reserved | perfectionistic |
| suave | restless | aggressive | undecided |
| argumentative | clownish | naïve | unconventional |
| submissive | daredevil | self-righteous | conformist |
| conforming | discontented | crafty | anxious |
| unstudious | impulsive | prideful | skeptical |
| moralistic | subtle | indecisive | changeable |
| silent | strict | spendthrift | middleclass |
| tense | authoritative | self-possessed | normal |
| self-conscious | average | choosy | critical |
| ordinary | meek | sensitive | blunt |
| emotional | uninquisitive | lonely | cunning |
| timid | forgetful | withdrawing | dependent |

*Note.* The list of word stimuli (personality/trait adjectives) were drawn from Anderson et al. (1968). Words were matched on valence and number of vowels. A and B lists were counterbalanced between participants.

**Supplementary Results**

**Table S3**

*Significant Task-Induced Suppression Associated with Each Task*

|  | Coordinates | | |  |  |  |
| --- | --- | --- | --- | --- | --- | --- |
| Regions | x | y | z | Cluster Size | Peak T | Z |
| *Rest > Reappraise* |  |  |  |  |  |  |
| Posterior insular cortex (R) | 44 | -16 | -2 | 5883 | 14.62 | >14 |
| Posterior insular cortex (L) | -42 | -18 | -2 | 4316 | 12.66 | >12 |
| Posterior cingulate cortex (R) | 4 | -30 | 44 | 6694 | 11.32 | >11 |
| Anterior cingulate cortex (L) | 0 | 42 | -6 | 1891 | 9.64 | > 9 |
| Middle frontal gyrus (R) | 28 | 30 | 32 | 112 | 4.30 | 4.00 |
| Dorsolateral prefrontal cortex (R) | 0 | -50 | -42 | 34 | 4.16 | 3.96 |
| Anterior cingulate cortex (R) | 2 | -12 | -26 | 39 | 3.96 | 3.78 |
| Post-central gyrus (R) | 40 | -16 | 38 | 99 | 3.94 | 3.76 |
| Superior frontal gyrus (R) | 2 | -30 | -50 | 19 | 3.59 | 3.45 |
| Pre-central gyrus (R) | 18 | -28 | -40 | 21 | 3.50 | 3.38 |
| Superior frontal cortex (R) | 24 | 16 | 50 | 18 | 3.48 | 3.35 |
| Middle frontal gyrus (L) | -28 | 30 | 34 | 21 | 3.30 | 3.19 |
| Middle frontal gyrus (R) | 28 | -46 | -30 | 12 | 3.15 | 3.05 |
| *Rest > Face-Matching* |  |  |  |  |  |  |
| Precuneus (L) | -12 | -62 | 16 | 45129 | 14.88 | >14 |
| Middle occipital cortex (L) | -40 | -76 | 32 | 583 | 8.81 | 7.39 |
| Middle frontal gyrus (L) | -22 | 30 | 38 | 1423 | 8.11 | 6.95 |
| Anterior insular cortex (R) | 36 | 4 | 10 | 123 | 7.40 | 6.47 |
| Anterior insular cortex (L) | -34 | 2 | 10 | 101 | 6.95 | 6.16 |
| Middle occipital cortex (R) | 44 | -74 | 32 | 150 | 5.40 | 4.99 |
| Cerebellum (R) | 24 | -58 | -36 | 56 | 3.99 | 3.81 |
| Inferior temporal gyrus (L) | -42 | -8 | -28 | 72 | 3.50 | 3.37 |
| *Rest > Letter-Discrimination* |  |  |  |  |  |  |
| Precuneus (R) | 14 | -58 | 18 | 67649 | 16.94 | >16 |
| Anterior insular cortex (R) | 34 | 4 | 10 | 66 | 6.09 | 5.53 |
| Inferior frontal gyrus (R) | 52 | 32 | 4 | 89 | 3.94 | 3.77 |
| Thalamus (R) | 0 | -14 | 2 | 22 | 3.34 | 3.23 |
| Orbitofrontal cortex (R) | 0 | 24 | -28 | 18 | 3.22 | 3.12 |
| Inferior temporal gyrus (R) | 26 | -6 | -46 | 58 | 3.21 | 3.11 |
| Cerebellum (R) | 14 | -36 | -32 | 14 | 2.67 | 2.61 |

*Note.* Significant task-induced suppression associated with the primary contrast of interest for each task. Coordinates (peak voxel) are in MNI space (mm). Cluster size = # of voxels (> 10 continuous). Magnitude and extent statistics correspond to a minimum threshold of *P*_FDR_ < .05. Peak T & Z = SPM T-score and Z-score statistics. L = left hemisphere; R = right hemisphere.

**Figure S4**

*Distinct* *Task-Induced Suppression Associated with Each Task*


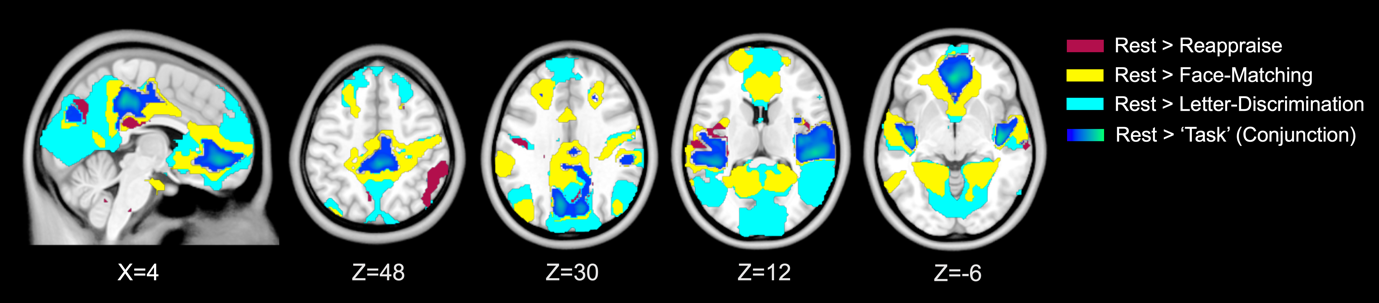


*Note.* Overlay of distinct task-induced suppression associated with each task: ‘Rest > Reappraise’ (in magenta); ‘Rest > Face-Matching’ (in yellow); ‘Rest > Letter-Discrimination’ (in cyan), and conjunction map showing the common suppressed regions associated with all tasks (see also Figure 1B).

**Regional Dynamic Activity Time-Series Analysis**

In an exploratory analysis we wanted to examine the consistency of the task evoked neural dynamics in the common peak suppression clusters in each task: medial prefrontal cortex (MPFC), dorsal posterior cingulate cortex (dPCC), precuneus, posterior insula (pINS), and frontal eye fields (FEF). Group-level (model predicted) responses for these regions for each task are presented in Figure S5. The results endorse the consistency of the task evoked neural dynamics as broadly highlighted by the GLM findings. Namely, we noted a consistent pattern of responses in the MPFC showing increased activity during rest and sustained suppression during task conditions which was more pronounced in the attentionally demanding blocks, and most notably apparent in the self-referential processing task. This is consistent with the standard DMN view. Interestingly, we also noted a consistent and distinct coupling between the MPFC and the pINS across the three tasks. Regarding the dPCC, precuneus and FEF, we noted a consistent pattern of response switches between rest and task conditions which is more noticeable in the self-referential processing task.

**Figure S5**

*Regional Dynamic Activity Time-Series for Each Task*


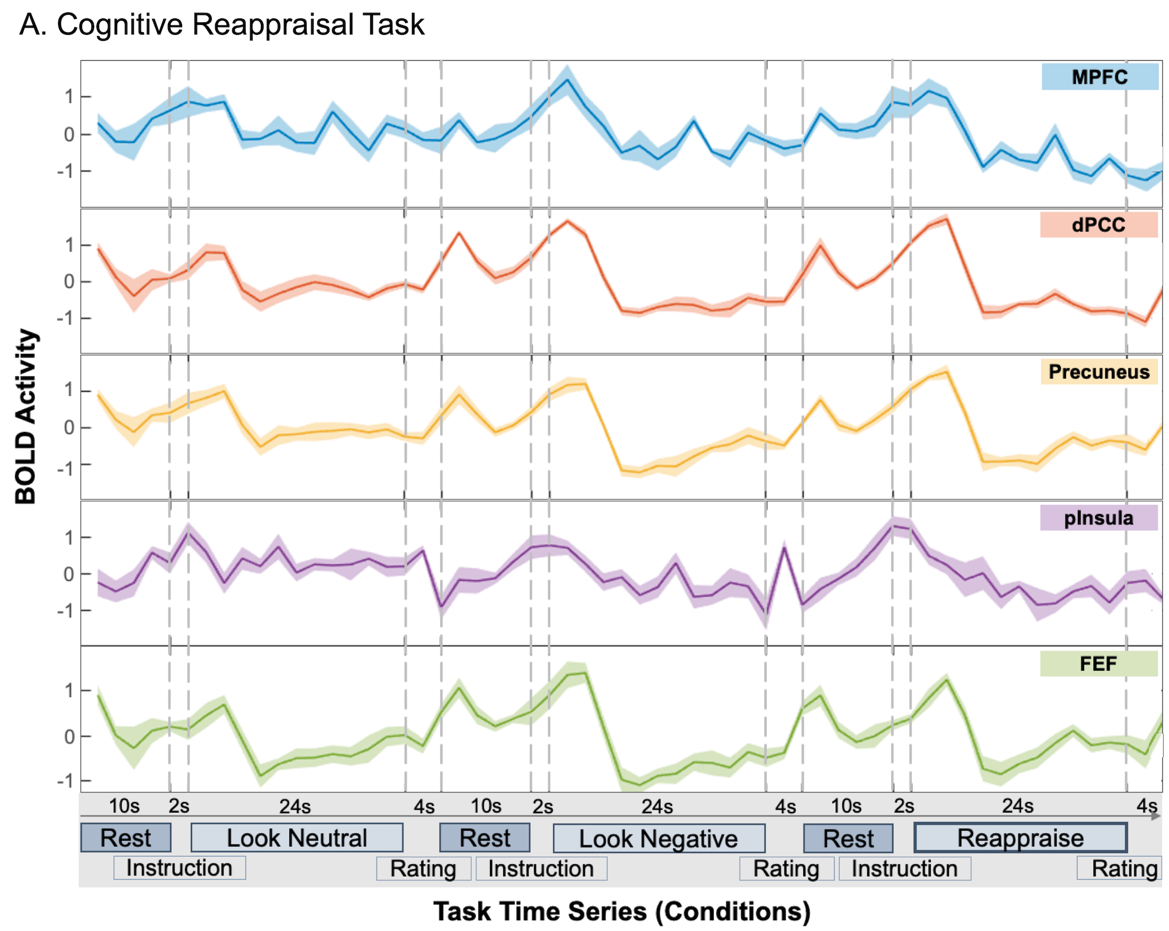


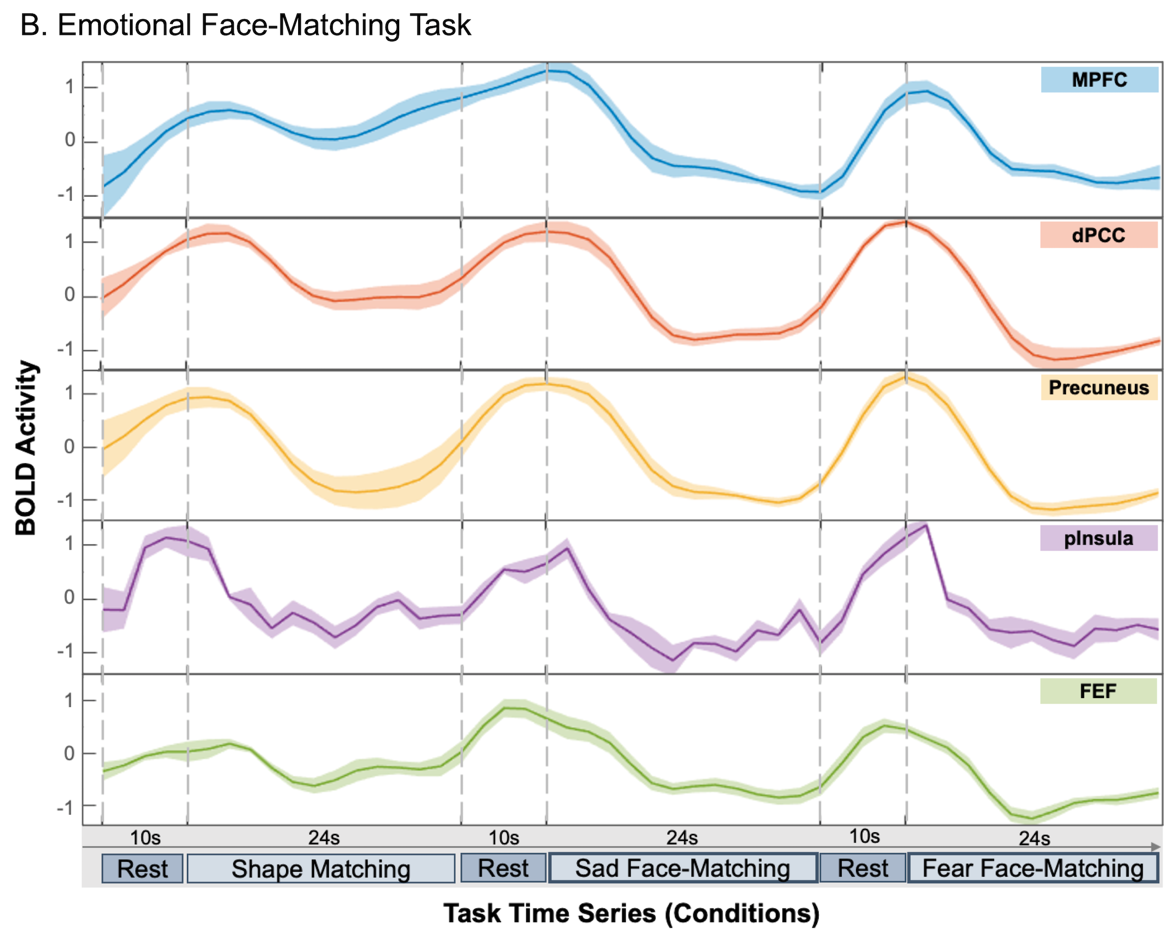

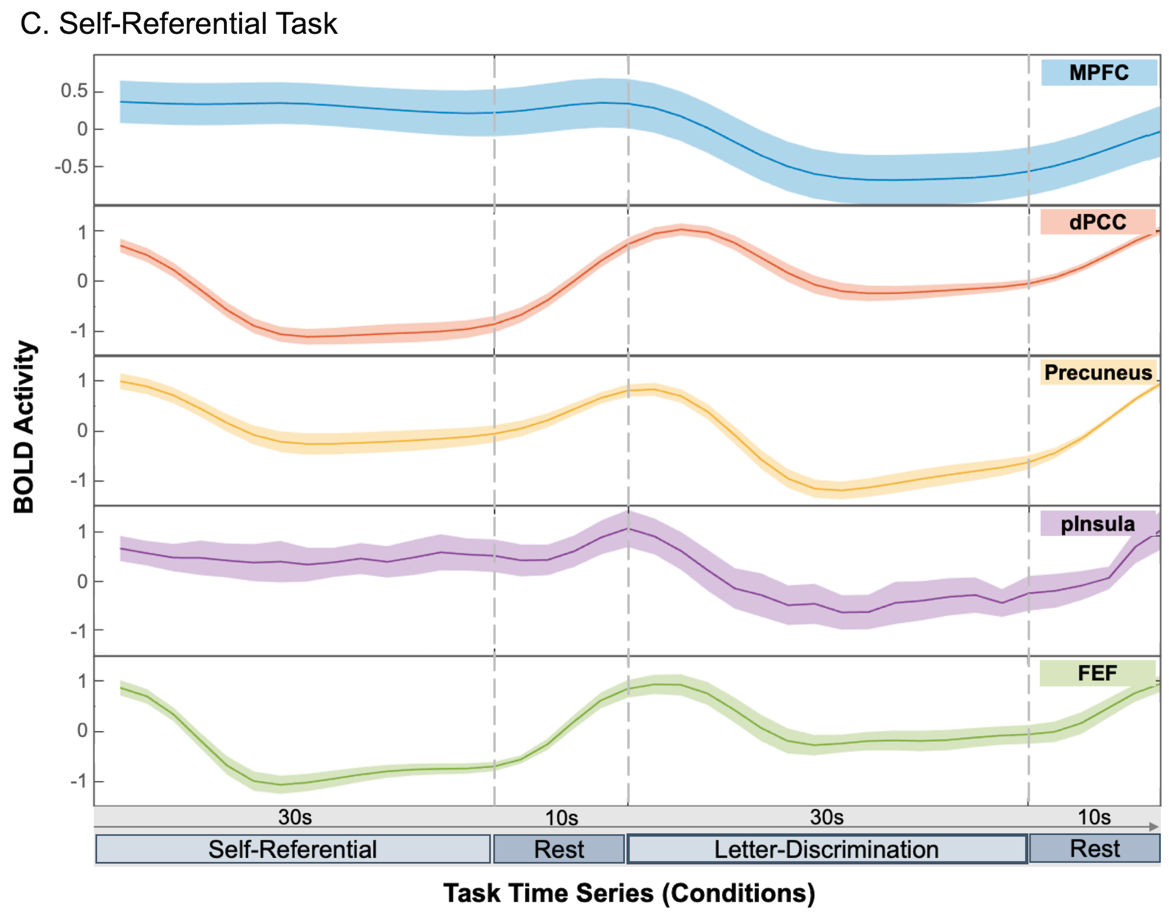


*Note.* Regional dynamic activity of group level responses across the entire sequence of each task for five regions-of-interest: MPFC (medial prefrontal cortex) in blue; dPCC (dorsal posterior cingulate cortex) in red; Precuneus in yellow; pINS (posterior insula) in purple; & FEF (frontal eye fields) in green. Shading represents standard error of the mean (SEM). X-axis = task sequence time in seconds (s); Y-axis = estimated BOLD signal change (arbitrary units).

**Figure S6**

*Cross-Task Pairwise Correlations of Task-Induced Suppression Within Individuals*


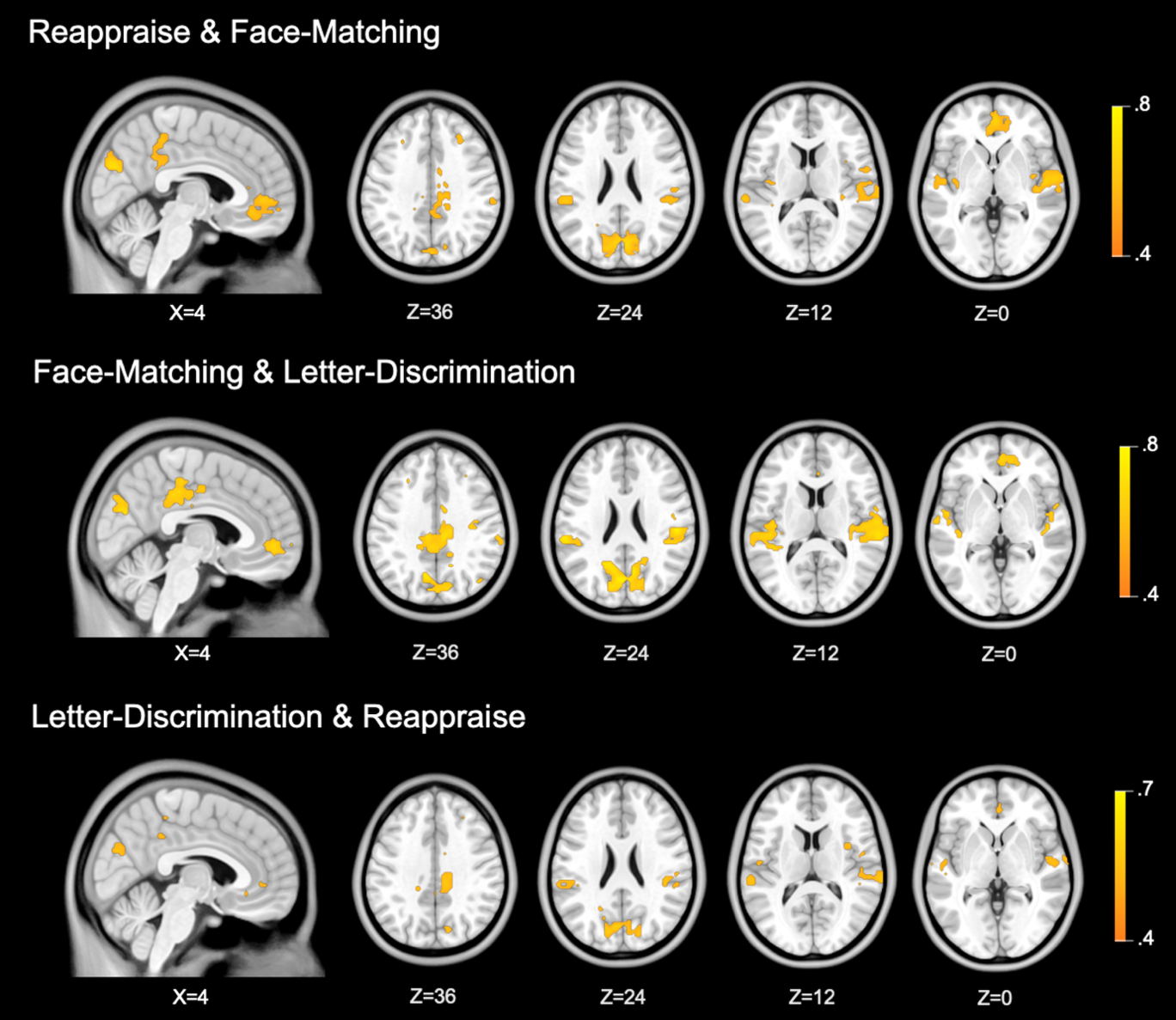


*Note.* Cross-task pairwise correlations showing significant within-subject correlated suppression maps between task-pairs across the three tasks: ‘Rest>Reappraise & Rest>Face-Matching’; ‘Rest>Face-Matching & Rest>Letter-Discrimination’; ‘Rest>Letter-Discrimination & Rest>Reappraise’. Displayed contrast maps are BPM correlations with a statistical threshold of *r* > .4. Colorbar represents correlation statistics.

**Table S4**

*Cross-Task Pairwise Pearson’s Correlations of Task-Induced Suppression*

|  | 1. | 2. | 3. | 4. | 5. | 6. | 7. | 8. | 9. |
| --- | --- | --- | --- | --- | --- | --- | --- | --- | --- |
| 1. Reapp_rACC | - | .63^*^ | .45^*^ | - | - | - | - | - | - |
| 2. Faces_rACC | - | - | .63^*^ | - | - | - | - | - | - |
| 3. Letter_rACC | - | - | - | - | - | - | - | - | - |
| 4. Reapp_dPCC | - | - | - | - | .59^*^ | .46^*^ | - | - | - |
| 5. Faces_dPCC | - | - | - | - | - | .62^*^ | - | - | - |
| 6. Letter_dPCC | - | - | - | - | - | - | - | - | - |
| 7. Reapp_PreC | - | - | - | - | - | - | - | .63^*^ | .60^*^ |
| 8. Faces_PreC | - | - | - | - | - | - | - | - | .73^*^ |
| 9. Letter_PreC | - | - | - | - | - | - | - | - | - |

*Note.* Cross-task pairwise Pearson’s correlations of task-induced suppression: [‘Rest>Reappraise & Rest>Face-Matching’; ‘Rest>Face-Matching & Rest>Letter-Discrimination’; ‘Rest>Letter-Discrimination & Rest>Reappraise’] in the three main suppression clusters identified from the regional overlap. Reapp = reappraise condition (cognitive reappraisal task); Faces = face-matching condition (emotional face-matching task); Letter = letter-discrimination condition (self-referential processing task). rACC = rostral anterior cingulate cortex. dPCC = dorsal posterior cingulate cortex. PreC = precuneus *P* < .05; * = *p* <.001.

**Table S5**

*Cross-Task Pairwise BPM Correlations for Each Task-Pair*

|  | Coordinates | | |  |  |  |
| --- | --- | --- | --- | --- | --- | --- |
| Regions | x | y | z | Cluster Size | Peak R | Z |
| *Reappraise & Face-Matching* |  |  |  |  |  |  |
| Occipital pole (R) | 0 | -82 | 28 | 913 | .85 | >.8 |
| Posterior insular cortex (R) | 52 | -12 | -2 | 1073 | .72 | 7.66 |
| Anterior cingulate cortex (R) | 2 | 48 | 0 | 672 | .67 | 6.96 |
| Middle frontal gyrus (R) | 26 | 34 | 32 | 48 | .65 | 6.69 |
| Posterior cingulate cortex(R) | 10 | -36 | 46 | 602 | .61 | 6.17 |
| Inferior frontal gyrus (L) | -60 | -34 | 8 | 91 | .61 | 6.15 |
| Insular cortex (R) | 42 | 0 | -18 | 20 | .60 | 6.07 |
| Precentral gyrus (R) | 52 | -4 | 18 | 60 | .59 | 5.88 |
| Inferior temporal gyrus (L) | -54 | -36 | 24 | 120 | .58 | 5.73 |
| Posterior insular cortex (L) | -50 | -12 | 0 | 120 | .53 | 5.18 |
| Insular cortex (L) | -42 | -14 | 14 | 84 | .52 | 5.12 |
| Posterior cingulate (L) | -14 | -42 | 40 | 19 | .52 | 5.06 |
| Anterior cingulate cortex (L) | -4 | 30 | -14 | 40 | .51 | 4.97 |
| Insular cortex (R) | 36 | 2 | 8 | 11 | .51 | 4.93 |
| Posterior cingulate (L) | -20 | -60 | 26 | 11 | .51 | 4.90 |
| Primary somatosensory cortex (R) | 28 | -38 | 68 | 12 | .50 | 4.78 |
| Cingulate cortex (R) | 10 | 0 | 36 | 12 | .49 | 4.74 |
| Dorsolateral prefrontal cortex (L**)** | -30 | 30 | 34 | 10 | .49 | 4.71 |
| Superior frontal gyrus (R) | 26 | -40 | 54 | 64 | .48 | 4.65 |
| Pre-central gyrus (R) | 12 | -18 | 48 | 10 | .46 | 4.43 |
| Insular cortex (L) | -42 | -22 | -8 | 11 | .45 | 4.29 |
| Posterior cingulate cortex (L) | -8 | -26 | 38 | 19 | .45 | 4.28 |
| *Face-Matching & Letter-Discrimination* | |  |  |  |  |  |
| Inferior temporal gyrus (R) | 52 | -24 | 28 | 1560 | .79 | >.8 |
| Posterior cingulate (L) | -16 | -60 | 28 | 2269 | .78 | >.8 |
| Inferior temporal gyrus (L) | -62 | -30 | 24 | 665 | .71 | 7.61 |
| Paracingulate gyrus (R) | 8 | 50 | -4 | 416 | .65 | 6.67 |
| Cingulate cortex (R) | 10 | 0 | 36 | 10 | .62 | 6.24 |
| Middle frontal gyrus (R) | 26 | 34 | 32 | 32 | .58 | 5.74 |
| Middle frontal gyrus (L) | -26 | 32 | 32 | 21 | .56 | 5.59 |
| Middle temporal gyrus (L) | -62 | -12 | -2 | 18 | .56 | 5.51 |
| Middle frontal gyrus (R) | 36 | -16 | 34 | 27 | .54 | 5.26 |
| Anterior cingulate cortex (R) | 2 | 32 | 12 | 20 | .50 | 4.89 |
| Posterior insular cortex (R) | 40 | -6 | -10 | 17 | .49 | 4.73 |
| Post-central gyrus (R) | 18 | -42 | 70 | 17 | .47 | 4.52 |
| *Letter-Discrimination & Reappraise* | |  |  |  |  |  |
| Posterior cingulate (R) | 18 | -76 | 32 | 574 | .68 | 7.09 |
| Middle temporal gyrus (R) | 70 | -28 | 12 | 435 | .61 | 6.20 |
| Inferior frontal gyrus (R) | 24 | 32 | 12 | 13 | .60 | 5.98 |
| Occipital pole (R) | 16 | -80 | 30 | 67 | .58 | 5.76 |
| Inferior temporal gyrus (R) | 46 | -28 | 26 | 83 | .58 | 5.75 |
| Superior temporal gyrus (L) | -62 | -30 | 14 | 127 | .58 | 5.75 |
| Posterior cingulate cortex (R) | 10 | -30 | 36 | 204 | .57 | 5.70 |
| Insular cortex (R) | 36 | 2 | 8 | 31 | .55 | 5.41 |
| Post-central gyrus (L) | -20 | -42 | 52 | 19 | .52 | 5.03 |
| Post-central gyrus (R) | 30 | -34 | 58 | 41 | .50 | 4.87 |
| Posterior cingulate cortex (L) | -16 | -36 | 36 | 36 | .49 | 4.78 |
| Pre-central gyrus (R) | 52 | -2 | 12 | 13 | .49 | 4.77 |
| Anterior cingulate cortex (R) | 2 | 44 | -2 | 22 | .49 | 4.72 |
| Superior temporal gyrus (L) | -42 | -40 | 18 | 27 | .49 | 4.69 |
| Posterior insular cortex (L) | -54 | -10 | 4 | 58 | .47 | 4.49 |
| Anterior frontal gyrus (R) | 14 | 50 | -8 | 12 | .46 | 4.40 |

*Note.* Cross-task significant pairwise correlations between each task-pair: [‘Rest>Reappraise & Rest>Face-Matching’; ‘Rest>Face-Matching & Rest>Letter-Discrimination’; ‘Rest>Letter-Discrimination & Rest>Reappraise’]. Coordinates (peak voxel) are in MNI space (mm). Cluster size = # of voxels (> 10 continuous). Magnitude and extent statistics correspond to a statistical threshold *r* > .4. Peak R = BPM correlation coefficient from peak voxel. Z = SPM Z-score statistics. L = left hemisphere; R = right hemisphere.

**Table S6**

*Descriptive Statistics for the Suppression Index, Demographic and Psychological Variables*

| Measure | N^†^ | Min | Max | Mean (SD) |
| --- | --- | --- | --- | --- |
| Suppression index | 84 | -0.0 | 1.0 | 0.5 (0.2) |
| Age | 84 | 15.1 | 25.9 | 20.0 (2.8) |
| IQ_WTAR | 84 | 22.0 | 49.0 | 42.3 (5.0) |
| IQ_FSIQ | 84 | 87.0 | 126.0 | 110.4 (8.0) |
| RRS | 84 | 10.0 | 35.0 | 17.6 (5.8) |

*Note.* IQ = Intelligence Quotient. WTAR = Wechsler test of adult reading raw score. FSIQ = Full scale IQ (transformed to age-adjusted standard scores from raw WTAR scores). RRS = ruminative responses scale (short version). **^†^**One participant was removed from the associated supplementary correlation analyses reported in the results as their suppression index was observed as being abnormally higher than the sample average.

**Supplementary Material**

References

Anderson, N. H. (1968). Likableness ratings of 555 personality-trait words. *Journal of Personality and Social Psychology*, *9*(3), 272. https://doi.org/10.1037/h0025907

Geday, J., Gjedde, A., Boldsen, A. S., & Kupers, R. (2003). Emotional valence modulates activity in the posterior fusiform gyrus and inferior medial prefrontal cortex in social perception. *Neuroimage*, *18*(3), 675-684. https://doi.org/10.1016/S1053-8119(02)00038-1

Hariri, A. R., Bookheimer, S. Y., & Mazziotta, J. C. (2000). Modulating emotional responses: Effects of a neocortical network on the limbic system. *NeuroReport*, *11*(1), 43–48. https://doi.org/10.1097/00001756-200001170-00009

Heatherton, T. F., Wyland, C. L., Macrae, C. N., Demos, K. E., Denny, B. T., & Kelley, W. M. (2006). Medial prefrontal activity differentiates self from close others. *Social Cognitive and Affective Neuroscience*, *1*(1), 18-25. https://doi.org/10.1093/scan/nsl001

Kelley, W. M., Macrae, C. N., Wyland, C. L., Caglar, S., Inati, S., & Heatherton, T. F. (2002). Finding the self? An event-related fMRI study. *Journal of Cognitive Neuroscience*, *14*(5), 785-794. https://doi.org/10.1162/08989290260138672

Lang PJ, Bradley MM, Cuthbert BN (2008): International affective picture system (IAPS): affective ratings of pictures and instruction manual. Technical Report A-8. Gainesville, FL: Center for Research in Psychophysiology, University of Florida.

Langner, O., Dotsch, R., Bijlstra, G., Wigboldus, D. H., Hawk, S. T., & Van Knippenberg, A. D. (2010). Presentation and validation of the Radboud Faces Database. *Cognition and Emotion*, *24*(8), 1377-1388. https://doi.org/10.1080/02699930903485076

McRae, K., Gross, J. J., Weber, J., Robertson, E. R., Sokol-Hessner, P., Ray, R. D., ... & Ochsner, K. N. (2012). The development of emotion regulation: an fMRI study of cognitive reappraisal in children, adolescents and young adults. *Social Cognitive and Affective Neuroscience*, *7*(1), 11-22. https://doi.org/10.1093/scan/nsr093

Moran, J. M., Macrae, C. N., Heatherton, T. F., Wyland, C. L., & Kelley, W. M. (2006). Neuroanatomical evidence for distinct cognitive and affective components of self. *Journal of Cognitive Neuroscience*, *18*(9), 1586-1594. https://doi.org/10.1162/jocn.2006.18.9.1586

Phan, K. L., Fitzgerald, D. A., Nathan, P. J., Moore, G. J., Uhde, T. W., & Tancer, M. E. (2005). Neural substrates for voluntary suppression of negative affect: a functional magnetic resonance imaging study. *Biological Psychiatry*, *57*(3), 210-219. https://doi.org/10.1016/j.biopsych.2004.10.030

Stephanou, K., Davey, C. G., Kerestes, R., Whittle, S., Pujol, J., Yücel, M., ... & Harrison, B. J. (2016). Brain functional correlates of emotion regulation across adolescence and young adulthood. *Human Brain Mapping*, *37*(1), 7-19. https://doi.org/10.1002/hbm.22905

Whitfield-Gabrieli, S., Moran, J. M., Nieto-Castañón, A., Triantafyllou, C., Saxe, R., & Gabrieli, J. D. (2011). Associations and dissociations between default and self-reference networks in the human brain. *Neuroimage*, *55*(1), 225-232. https://doi.org/10.1016/j.neuroimage.2010.11.048
